# Running an XR lab in the context of COVID-19 pandemic: Lessons learned from a Norwegian university

**DOI:** 10.1007/s10639-021-10446-x

**Published:** 2021-02-18

**Authors:** Jose Garcia Estrada, Ekaterina Prasolova-Førland

**Affiliations:** Norwegian University of Science and Technology NTNU, Trondheim, 7491 Norway

**Keywords:** XR lab, Covid-19, Immersive learning environments

## Abstract

Universities and companies were not prepared to the changes introduced to limit the spread of COVID-19 in Norway. Universities had to switch to online teaching overnight. There is still uncertainty how measures to control the pandemic will keep affecting universities in the short and middle term. Such measures have consequences on how to carry out research that usually relies on students, researcher and volunteers using the equipment and applications. Our group carries out research on virtual/augmented/extended reality (VR/AR/XR) for immersive training and learning. This research often involves user studies. We had established procedures on how to use the equipment, carry out demonstrations and teaching for students, teachers and visitors, develop projects as part of bachelor and master projects and test new applications with volunteers. The measures taken by authorities to control the spread of the pandemic made it difficult or unfeasible to carry out some of those activities. In this paper we describe how our group and XR lab reacted after universities were closed to students’ presence in campus in March 2020. We present our actions to keep research ongoing, our evaluation of some of those actions and discuss how we had to change the way we operate our XR lab in order to continue teaching and research in the near future, under the assumption that restrictions due to the pandemic can be re-implemented at short notice. We propose procedures to run an XR lab in a manner that inspires visitors to feel safe and confident of using the equipment. Our contribution is the proposal of procedures to run an educational XR lab safely and contribute towards the conversation about how to carry out research involving users in XR under pandemic restrictions.

## Introduction

A common denominator among many industrial and education organisations during the COVID-19 pandemic is that few anticipated the effects of government-imposed measures into the operation of businesses and education organisations. Those effects are ongoing and currently we can only have a partial reflection at best.

Norway adopted measures to limit the spread of the SARS-CoV-2 (Covid-19) in early March 2020. Those measures required the closure of universities, return of higher education students to their homes or confinement to residences, absence of physical presence lectures and work from home. Students attended classes remotely and took exams digitally at the end of the academic year 2019–2020.

There were also important effects on research. Many researchers have attended virtual conferences since March 2020 instead of being there in person. This new experience has been forced upon the research community by the need to move conferences online due to the unfeasibility of large gatherings and limitations in international travel. Most universities in Europe moved academics to work from home and classes were moved online. Experiencing events like IEEE VR 2020 as an online event with possibility of VR presence provided a case that illustrated the potential and challenges of VR for substituting physical attendance to events.

The disruptions were not limited to major academic events. Activities directly related to research also were affected. Our group carries out research on immersive environments (VR/AR/XR) for learning and training. Usability tests are important to evaluate the applications developed in research projects. The execution of studies requiring volunteers became challenging due to the restrictions on physical contact and meeting.

The challenges created by replacing physical presence to compulsory remote communication open new possibilities for XR-supported remote collaboration and learning. The unexpected present scenario requires refreshing learned lessons and review what has been successful to cope with the new demands on remote support.

Research in XR focuses on key aspects of the technology. Rarely there is work addressing the logistics and practicalities of presenting XR technology to public outside researchers directly involved with XR. The restrictions by authorities aimed at controlling the spread of the epidemic also can have long lasting effects in the ability to carry user studies in XR. In order to be able to continue research and education activities in XR labs in the context of the pandemic it is crucial to develop appropriate hygienic procedures and protocols.

This paper discusses our experiences with using XR for immersive learning research in higher education at a Norwegian university, running an XR lab during a national lockdown and the planning for the “new normal”. We contribute to the immersive learning and XR community by presenting best practices in operating a teaching, research and demonstration XR lab. We propose that the procedures we adopt enable keeping XR research and teaching active also during ongoing restrictions and provide a safe environment for the users of the lab.

## Related work

Technologies from extended reality offer complementary solutions to videoconferencing applications. Most of those solutions offer real-time communication, video and audio. However, the 2D display on a flat screen and size of displays are among factors that limit the sense of co-presence and the use of gaze, gesture, posture and nonverbal cues (Pejsa et al. 2016), something that could partly be alleviated by the use of immersive technologies.

XR technologies offer several possibilities for their use in education. An important proportion of publications in education and training using XR technologies focus on improving learning in natural science, history, computer science and mathematics (de Belen et al. 2019). Augmented reality can support learning while playing in primary school education. Learning gains and an effect in motivation are among the reported advantages of using AR systems in education (Garzón et al. 2019). Use of VR in education reports benefits for teaching procedural-practical knowledge, declarative knowledge, analytical/problem-solving skills, theoretical concepts and soft skills (Radianti et al. 2020).

Adoption of XR in education has to overcome some challenges such as access to equipment and content creation and availability. It is often commented on XR literature how devices have become more affordable (Rodriguez 2016; Manis and Choi 2019). Nonetheless, the technology is not yet mainstream (Heinonen 2017; Ghobadi and Sepasgozar 2020) and therefore is not common that individuals own XR devices. Usually they will use devices acquired by an employer or an organization with enough funding. Game creation engines and applications like SteamVR® facilitate content creation. Companies like Oculus, Microsoft and Valve offer XR content either free or paying. Some researchers also imply that more XR content needs to be developed (Ghobadi and Sepasgozar 2020). Additional challenges are, for example, providing users of immersive virtual environments with correct and sufficient awareness of other users (Xia et al. 2018). Nonverbal cues are often missing in remote collaboration solutions in XR. They are important since they can affect user performance even more than lack of verbal communication (Teo et al. 2019). (Jo et al. 2017) found that presenting avatars in a real/AR background helped to improve trust in collaboration and co-presence.

Universities are replacing face-to-face teaching with online and remote methods due to the changes in education prompted by the pandemic (Yusoff et al. 2020). This is the context in which we are increasing the use of XR for education and adapting to the new restrictions in human contact.

However, research in XR is also complicated by the pandemic. Some XR equipment is expensive even for researchers and not easily available. There are also concerns about reusing XR equipment that is used in close contact by a participant, for example in a study, and that may be difficult to sterilize (Steed et al. 2020).

## XR in a Norwegian city before restrictions in human- to human contact

Our research group and XR lab IMTEL (Innovative Immersive Technologies for Learning) carries out research and education in XR at the Norwegian University of Science and Technology, in the city of Trondheim in Norway. We see more companies developing and using XR in Trondheim. For instance, the Norwegian Labour and Welfare Administration (NAV abbreviation in Norwegian) in the city uses virtual reality applications for introducing jobseekers into some work opportunities. Several start-ups and industrial developers work on multiplayer VR games and VR/AR applications. Our lab interacts with many of those companies, sometimes holding events for them or inviting them to participate in events with or for students, industry and community.

Working from home (or home office as it is called in Norway) has been an opportunity for some XR developers to use more actively the tools of the trade. For example, a XR developing studio in Trondheim, BreachVR, used their VR platform for holding their remote meetings in March 2020 when all their employees were working from home. Nonetheless, before the pandemic, XR technologies have been used as technologies that can support different activities but the way in which they were used did not explicitly considered use in a pandemic context.

### Operating an XR lab

**Fig. 1:**
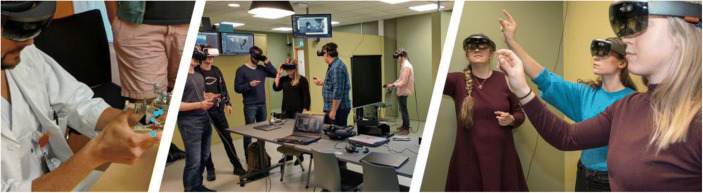
Examples of immersive learning activities within the IMTEL group

We have a dedicated XR lab to support staff and students in integrating XR into teaching in higher education. The lab is part of the (IMTEL) group**.** Our major activities are education, research and dissemination. We carry out immersive learning using HMDs and desktop applications. The XR lab is frequently used for teaching in different courses at our university. For example, geography students have used the lab as a part of their regular exercises on the topic of climate and experienced the results of climate change in VR with apps such as Stanford Ocean Acidification Experience (VHI Lab Stanford 2016) and Greenland Melting (Emblematic group 2019) as well as apps developed by IMTEL students visualizing sea level rise in the city of Trondheim. Students can also carry out their research projects in the lab for masters and bachelor programs. We work in cross-disciplinary groups with master and bachelor students from different departments, developers, teachers and involving stakeholders from the industry and public sector. The major goal of these activities is the design and co-creation of educational XR applications to be used in learning and training at our university, schools and other organizations. We also regularly arrange various dissemination events, receiving delegations from Norwegian schools and organizations, as well as international delegations. We have arranged a series of Innovation days, with talks from national and international guest speakers on various topics within Immersive Learning, with the possibility for the university students and employees and general public to visit the lab for demos and hands-on Fig. 1.

**Fig. 2:**
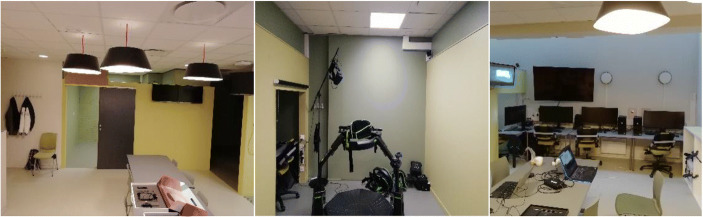
XR lab with common area and four dedicated rooms for HMDs

We have a 76 square meters dedicated lab. The lab has a main common area with meeting table, workstations and four rooms with a dedicated HMD using base stations, including one with a Virtuix Omni treadmill (Fig. 2) and a variety of other XR devices (Table 1).

**Table 1:** List of XR devices available at the lab

Description	quantity
Virtuix Omni treadmill	1
VR desktops	7
VR laptops	6
HTC Vive headsets	5
Mixed reality headsets	7
AR headsets	5

An important aspect of operating an XR lab is offering demonstrations of applications to visitors and colleagues. Normally the number of visitors exceeds the quantity of headsets available. Visitors will use the headsets taking turns. It is important to provide them with a headset that is appealing to use, clean and free from sweat from previous users. For those purposes, disinfection practices have been used in the lab for a long time. Those practices include cleaning headsets between users, using antibacterial wipes and visor covers in cleanable materials. Those procedures were predominantly aimed at demonstrations and external visits.

## Supporting the continuation of XR projects during pandemic and associated social restrictions

On March 12, 2020 the Norwegian government announced the suspension of physical presence of students at institutions of higher education. Educations was moved online, and work was to be done remotely. At the time there was uncertainty for how long these measures would be in place, therefore it was reasonable to worry about compromising the completion of research projects. The initial problem was that students were no longer allowed into campuses or laboratories. Therefore, they were unable to use XR equipment. An additional factor for students was the inability to carry out usability studies with volunteers.

### Reacting to short notice closure of university

Many universities around the world had to close suddenly. In many cases this allowed for little or no preparation for teachers, students and researchers. In our case, the university followed government regulations and informed students and staff to work from home with effect at the end of the day. This required that we reacted to enable students to complete their research work.

The lab leader prepared and implemented a response plan to continue support for students and their projects. Students were contacted via email and through collaboration app Slack to facilitate collaboration and communication. Equipment was assigned to each student according to their projects (Table 2).

**Table 2:** List of XR equipment loaned by the lab to students

Device	Quantity
MSI Leopard 8RF 64 bit	3
HP Omen 64 bit	1
HoloLens 1 AR headset	1
Magic Leap AR headset	2
HP Reverb VR	1
VIVE Cosmos	1
Oculus Quest 1	2
HP Windows mixed reality headset	2
HTC VIVE	1
Oculus S	1
Oculus Rift	3

It was also important to hand in the equipment in a safe way in order to avoid potential health risks or contravention of the government rules. The available equipment has been distributed between the students and student groups in accordance with the nature of their projects, time frame and equipment availability. For example, if 5–7 students worked together on the same assignment, only 1 headset and 1 VR-compatible PC was assigned to the group, mostly to the lead developer. The rest of the group would contribute to the development remotely using emulators and various tools for supporting remote work. If e.g. 2 master students worked on a project requiring the use of collaborative VR, they got assigned 2 sets of VR headset/laptop.

Time slots were assigned to students so they could come to collect their devices from the lab with half an hour interval between each other. This time difference was meant to eliminate the possibility of having students run into each other, thereby avoiding meeting in groups. It also helped to avoid students needing to use the same public transportation vehicle. One of the important government advices at the time were to reduce the number of passengers using public transportation. Equipment was disinfected before handing it over.

The equipment was delivered back in similar manner after the completion of the student project, being left in or just outside the lab or in designated places agreed on with IMTEL staff.

**Fig. 3:**
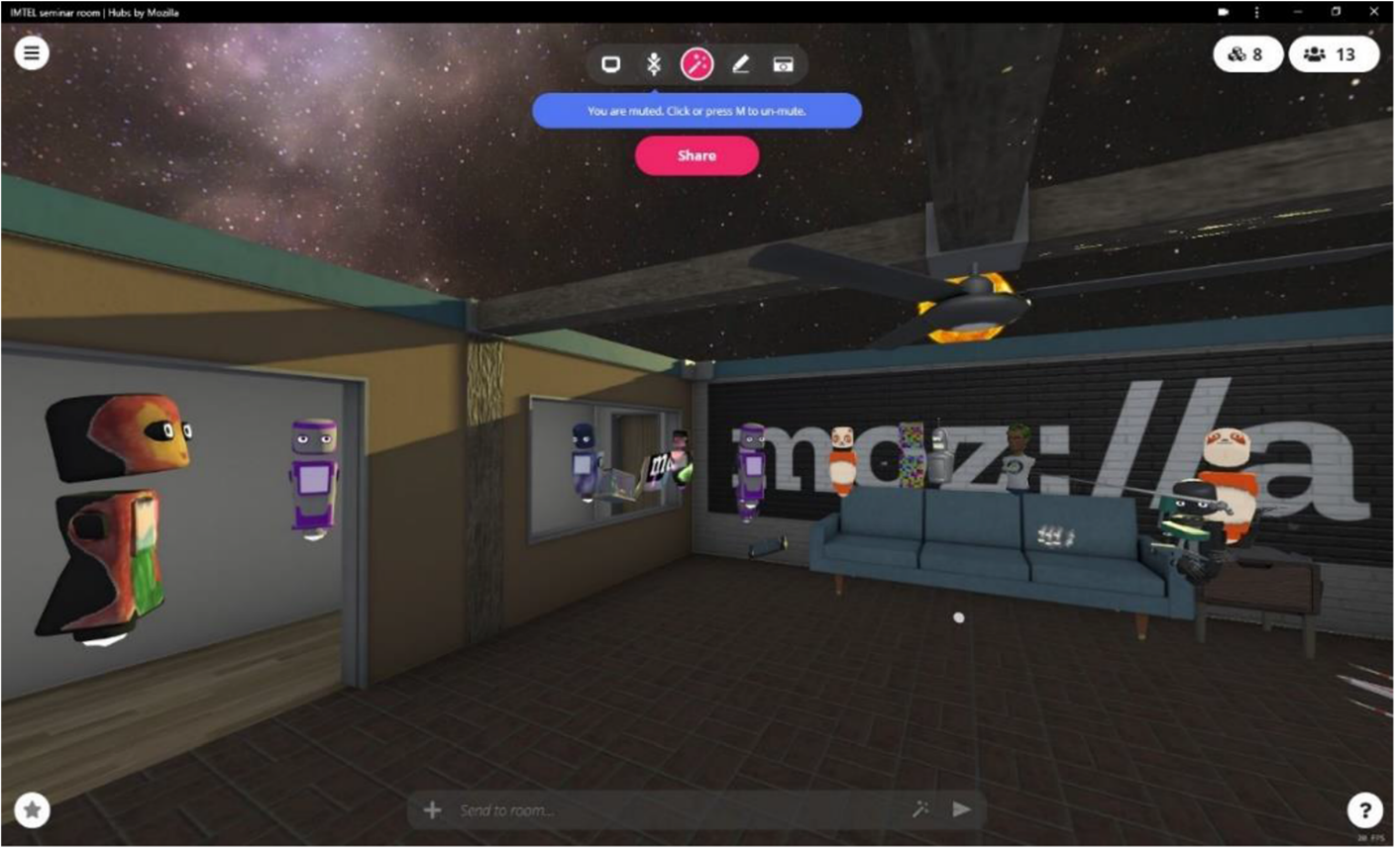
Lab meetings during lockdown in Mozilla Hubs

Students took the equipment home and were able to complete their projects. During the lockdown, we held virtual lab meetings and events, e.g. in Mozilla hubs (Fig. 3), to provide students with support on guidance on completing their projects in these unprecedented circumstances. All the students who borrowed the equipment were encouraged to run user tests for each other’s projects. We arranged a simple online document where students could publish requests for tests and volunteer as test persons. Additional test persons have been recruited from national contacts and collaborators and international XR communities as a short-term solution (Steed et al. 2020). In some cases, to reach a test audience of significant size, a video of the projects has been distributed on social media and through other channels together with a questionnaire, so that also potential users without access to the headsets could provide feedbacks. For some projects requiring use of specialized non-movable equipment (Virtuix OMNI treadmill), the tests had been performed by one of the IMTEL researchers who had access to the lab, with the students remotely conducting the test through TeamViewer or similar.

In late summer, the Norwegian government relaxed the restrictions on meeting and mobility. The students did not return to the campus for the remaining of the academic year 2019/2020.

The experience raised important questions about how we could operate the XR lab in the future considering that there is no clarity about the pandemic. We assumed that our reaction plan has been successful and also expected that we needed to change our operational procedures for the new academic year if there was not a clear solution to the pandemic crisis. One way to inform our decision making into the new academic year was to evaluate our reaction to the lockdown.

### Evaluating the response

We carried out a short survey to evaluate the experience of students using the XR lab and the impact of the pandemic limitation measures on their academic work. The survey aimed to gain an impression of the impact of the pandemic in the students working with immersive technologies in our university. The pandemic, as a rare and unexpected event, can have unexpected consequences in normal activities. Therefore, it is of importance to gain as much insight on its consequences, challenges and opportunities.

The number of students using immersive technologies for their research projects is significantly small compared to the student population in campus. Nonetheless, the nature of XR technologies are that there are fewer active users in comparison to widespread technologies. This is the reason why it is relevant to acquire data regarding this period and reflect on the experiences that XR groups and users have been through.

#### Short survey

The survey was carried out online among student population working at IMTEL lab**.** The condition for participating were to be a student working with immersive learning technologies for their master’s project or in a bachelor program.

Nineteen questions were presented to participants, divided in two sections. The survey form specified to reflect on their experience before and after the lockdown. The survey was designed to gather data that enables a comparison of the student’s perception of their XR projects before and after the lockdown measures. Two main traits are measured in the questionnaire: equipment availability and ability to dedicate time to the project.

XR equipment was kept in the IMTEL laboratory in the university. Before the pandemic, the students had no limitation in time to use XR equipment, but it was rarely lent to take home. Following the lockdown, the university instructed students to stay home and physical lectures were moved online. Effectively, students had no physical access into campus buildings between early March and early June, except for shorter visits required to pick up and later deliver the equipment.

### Participants

We conducted a short survey with master’s and bachelor students on July 2020. The survey was conducted after the students had completed their projects and submitted. Nine students replied to the survey, 6 of them were masters students. There were 14 students who needed XR devices during the period of the lockdown. Apart from the survey, we report qualitative observations and reflections from being XR student supervisors and teachers during the pandemic.

While the students who used the lab in their projects before the lockdown come from various disciplines, e.g. pedagogy, psychology, physics and biology, those who borrowed the equipment from the lab after the lockdown mostly studied IT, except from one student who studied business.

## Results

The 19 questions were scored on a 5-point Likert scale. The questions collected impressions from students on the impact of university’s closure on using XR equipment, compared to before closure as well as the impact in their overall project.

Answers are scored between 1 (strongly disagree) to 5 (strongly agree). The results of the survey suggest that students had a positive attitude towards their projects before the lockdown (Table 3).

**Table 3:** Questions related to XR equipment and project progress before lockdown

		mean	S.D
Limitations due to XR equipment			
Q1	I had access to the devices needed to do my work	4.78	0.42
Q2	I could work on my project at good pace with the devices I had	4.67	0.47
Q3	I was already using the devices needed for my work	4.78	0.42
Q4	I was waiting for devices being bought in order to advance on my project	1.33	0.67
Project management			
Q5	I had all I needed to complete my project	4.38	0.48
Q6	My project was within schedule	4.00	0.5
Q7	I had planned to do most work towards the end of the year anyway	2.5	1.22
Q8	My project was not dependent on having immersive technologies hardware	1.38	0.48
Q9	We had adequate support before the university closed	4.38	0.48

**Fig. 4:**
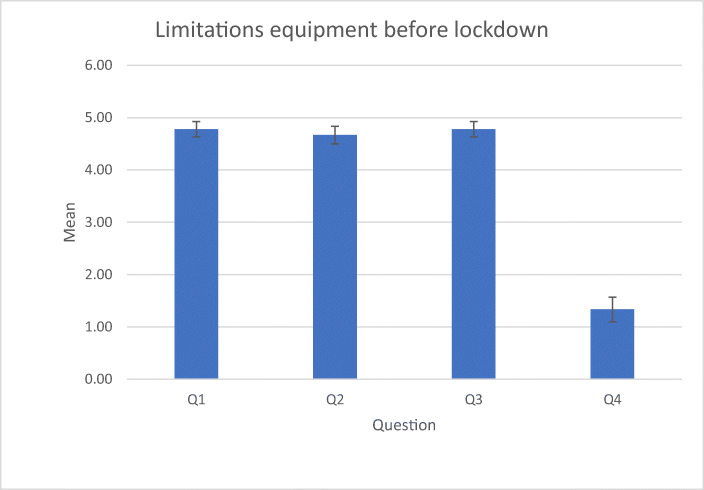
Answers to the first four questions

Answers (Fig. 4) suggest that the students had the technical equipment they needed. This is naturally expected and serves as a baseline to discuss their perception after the lockdown.

**Fig. 5:**
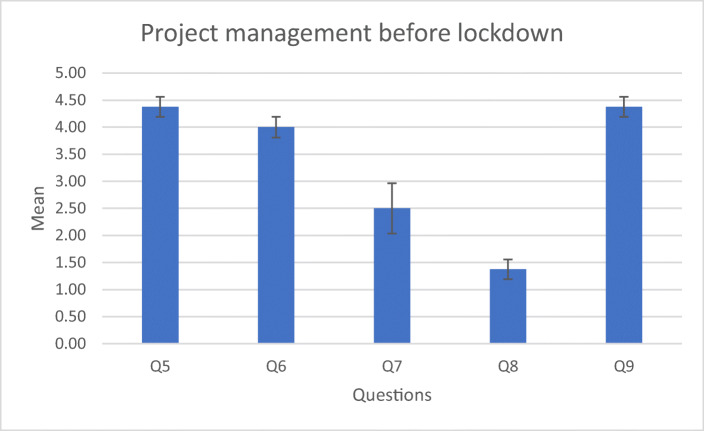
Answers to the project questions before lockdown

The answers (Fig. 5) also suggest that students did not consider that there were major problems with their project’s progress before the lockdown.

The questions after lockdown also aim to give insight into whether the students received the necessary support, under the circumstances (Table 4).

**Table 4:** Questions related to XR equipment and project progress after lockdown

		mean	S.D
Limitations due to XR equipment			
Q10	I had access to the devices needed to do my work	3.55	1.25
Q11	I was able to borrow a device shortly after university closed	4.66	0.47
Q12	Availability of devices did not delay my work	3.77	1.13
Q13	There were no devices available for me to continue my work	1.44	0.68
Project management			
Q14	I had all I needed to complete my project	3.13	1.17
Q15	Availability of devices did not delay my work	3.25	1.39
Q16	My project only required small adjustment after university closed	2.25	1.2
Q17	I had completed most of my work before the university closed	2.37	1.11
Q18	My project was not affected by availability of immersive technology devices	2.13	1.17
Q19	We had adequate support after the university closed	3.75	1.09

**Fig. 6:**
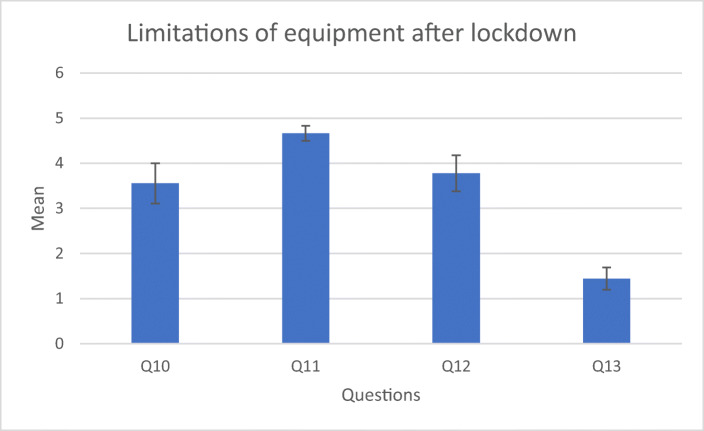
Attitude towards equipment after lockdown

Q10 and Q11 (Fig. 6) suggest a slight contradiction. Students agree that they were able to borrow equipment but are less positive on having all the devices needed for their work. A possible explanation is that some students used additional hardware apart from HMDs which are too bulky to be transported home. They might also refer to having a less powerful but portable PC.

The students seem to agree in their majority that they were provided with the devices they needed to continue their work.

Q14 is interesting because it showed a neutral attitude towards their ability to complete their project. In some cases, the projects were aiming to use volunteers to test and collect data on applications developed. That was no longer possible after lockdown.

**Fig. 7:**
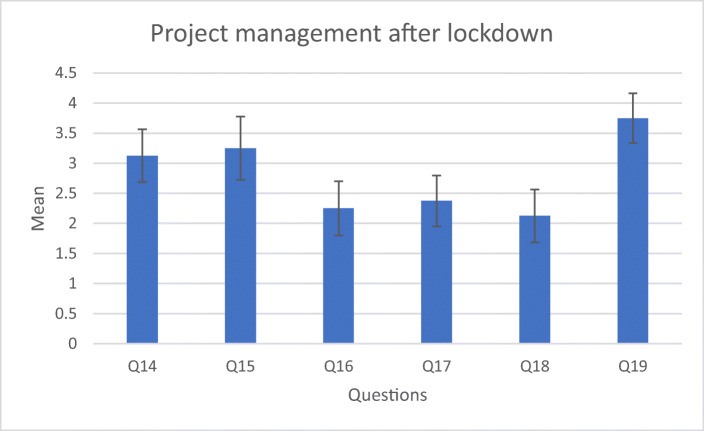
Attitude towards their project progress after lockdown

The answers (Fig. 7) suggest that students feel that the lockdown affected their projects and acknowledge they were supported. It is likely that this perception of support must also be interpreted considering the change between physical support and remote support.

It must be emphasised that students still had other assignments and courses being delivered online. In some cases, this could be a factor in their perception of project progress.

Qualitative observations from a teacher perspective can be summarized as follows:

- In general, the students highly appreciated the possibility to borrow the equipment as it was instrumental in finishing their projects as planned.
- The students in their 3rd and 4th year completed the projects on time or with about a week’s delay. The requirements for user testing in these projects were less than in last year’s master projects. In some cases, the user testing for this group was substituted with a description of planned test procedure or small-scale testing with persons from the same household.
- Most of the final year master students had to deliver with a significant delay (a month or more) after application to the university administration. The delay was mostly due to difficulties with access to test persons, but in some cases was also caused by mental health issues as a result of isolation during the lockdown.
- In the projects where students worked in groups of 5–7, some of the students expressed frustration with the disruption to face-to-face socializing and the suboptimal support provided by technologies for remote collaboration. The students used a combination of online VR platforms such as Mozilla Hubs and tools like Zoom and Blackboard for group meetings, socializing and presenting projects. Many reported technical problems resulting from suboptimal Wi-Fi coverage in dorms and limitations of personal laptops. As only one of the group members had access to the full VR set, the product development has been complicated. At the same time, all the groups managed to deliver final deliveries of high quality.
- XR students required additional and customized guidance during the lockdown. This included in particular how to address learning goals and research questions within the new restrictions without compromising the overall quality of deliveries and reformulating of research questions in certain cases. Additional issues included mental health challenges and the overall logistics of the student projects, such as organizing equipment access and exchange with appropriate disinfection procedures, facilitating access to international experts for expert walk-through and organization of remote trials where possible.

## Discussion

Our aim with the study is to provide a record and evidence of the impact of the COVID-19 restriction in education using XR and running a research and education XR lab. The limited population in the survey points to a major dilemma for researchers in XR. XR is not yet a highly disseminated activity. A common criticism when presenting data from XR studies is sample size. One interesting question is how can we conduct research when human-to-human contact is severely restricted? In our case, we cannot claim statistical significance with such a small population as we have. At the same time, many XR research groups and labs have a very limited number of users in comparison to the general student population of their institutions. Usually labs conduct studies with participants recruited among general student population, most of them without experience in XR.

The case we present here is one where we look at how students using immersive learning XR in their projects were affected. It is unlikely to find any group or lab that has large number of students using XR for their thesis and student projects. Nonetheless, we propose that this is a problem that deserves consideration due to its implication in the advance of XR research and immersive learning.

Therefore, together with the qualitative data, our research outlines some tendencies and allows to formulate some number of lessons learned for running an educational XR lab in the context of the pandemic. It has not been possible to carry out a systematic study since we have not expected this pandemic and were not able to plan pre-tests. Planning a similar study across several educational XR labs is complicated because of differences in institutions, hardware, teaching and research programs and data collection methods, though some early research on the topic already exists (Steed et al. 2020). One of the implications of this study is that we could encourage the XR community to come up with a strategy to combine their efforts to carry out more comprehensive studies.

Our students had difficulties since they have planned for conducting user tests with other students, which became difficult since they were not allowed to meet outside their households. Recruiting participants will become even more complex in pandemic settings. The core problem is how to control conditions when the experimenter is unable to be physically present or verify all conditions.

In some cases, some form of self-diagnostic will help to gather information on a participant’s PC, enabling the experimenter to verify technical settings. Experimenters will now have to consider using cameras to allow observation of the test, with ethical, security and privacy implications.

Nonetheless, research methods will have to change to adapt to this new situation. A question floated in conversations in the XR community is how to asses research carried out during this period. This question is of high importance if we consider that, at the time of writing this article, many countries seemed far from having the pandemic under control and questions still linger whether new spikes or waves are coming soon.

Our short survey and qualitative data support the idea that any XR group need to be able to hand equipment to students and researchers. However, it also shows that equipment is not the only limitation to XR projects.

### How we plan to use XR in post-pandemic situation

During the summer 2020 Norway presented low numbers of infections. The country re-opened its borders and in many aspects of society there was an apparent return to normal. However, the fall 2020 saw increased infection rates in Norway and the rest of the world. Norway and especially Trondheim was relatively mildly affected. The NTNU adopted a hybrid teaching model in the fall, so that NTNU’s campuses and our XR lab have been open to students and employees during with strict hygienic routines in place. Due to surge of infection rates towards the end of the fall, most of the teaching at NTNU (with the exception of lab work and similar activities requiring physical presence) was moved online. Despite the prospect of mass vaccination in the spring, the COVID-19 situation and the implications for campus operations remain unclear. Therefore, to be ready for several possible scenarios we have developed a preliminary strategy for running an educational XR lab in the context of the pandemic along the main strategy directions for our lab and research group: research, education and dissemination.

### Education

To adapt to the hybrid learning model that is the model used by our and several other European universities during the study year 2020–2021, we have introduced a number of measures. These measures are targeted at enabling social distancing among students and visitors at the XR lab, to provide students in quarantine and with symptoms to participate in classes virtually and to sustaining a high standard of hygiene at the lab.

We have used Mozilla Hubs for immersive learning since the spring 2020 lockdown to provide an additional platform for teaching and have also introduced online VR platforms such as Virbela and AltspaceVR into our repertoire. This has allowed us to deliver online and hybrid courses on XR as enabling technologies as a part of continuous education program, including a new course for those who lost their job as a result of the pandemic.

**Fig. 8:**
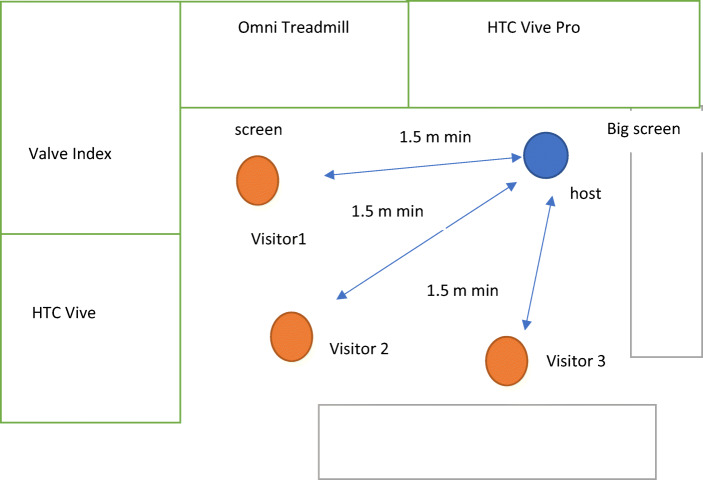
Position scheme for visitors. The common area is where visitors and host are illustrated

Our students working at the lab receive training in hygiene protocols, making sure of not sharing equipment without proper cleaning. Anyone feeling unwell to work is required to work remotely. We re-arranged the lab area so we can distribute students and visitors and keep distance between themselves and with the visit guide (Fig. 8). We limit visits to maximum three persons at the time in the common area of the lab, with maximum 11 persons at the lab at any point. We have physical separation in the rooms dedicated to HMDs so a person can be in any of them (probably with a helper) whilst three people can follow what they do on a screen.

We have developed a 3 step procedure for disinfection of XR equipment, in consultation with Norwegian Health authorities and international XR community: 1) Wiping the headsets and controllers with alcoholic wipes between users; 2) Use disposable HMD covers that provide a barrier between the skin and the headset; 3) Disinfecting the headsets in a UVC (Cleanbox) machine between uses. This allows to clean around the lenses on HMDs that are difficult to reach for cleaning manually. Furthermore, the students are instructed to keep 1-m distance at all times or wear a face covering in situations when it might be problematic.

### Research

We now faced the challenge to create methods to support remote collection of data. The challenges this approach brings are not trivial. One aspect is the practical implications of asking participants to participate in experiments from their homes. Guaranteeing data privacy in remote experiments will be more complicated when the experimenter can’t be physically there during the experiment. In the case of immersive environments research, we will need to deliver equipment to participants when there are restrictions for meeting. The disinfection routines we have developed (and described above) allow us to mitigate the constrains created by the risk of transmitting the virus by contact with shared objects. These routines also allow to receive limited number of test participants physically in the lab when the university regulations permit that.

The challenge for future research projects is to design projects in such a way they can be carried out with limited access to participants. There is an academic dimension, how to fulfil the academic requirements of the projects. An interesting question is how the scientific community will adapt the way they assess research considering that in many countries it will no longer be possible to have access to large number of participants for user evaluation and studies. It is likely that we need to come up with new methodologies to combine data from small groups in order to gain understanding of a problem at a larger scale since XR research groups are usually small and diverse. It is encouraging that many XR research groups have been communicating and volunteering to help as research subjects to other groups, even overseas.

### Dissemination

Dissemination and popularization of XR research has always been one of the top priorities of our lab. Prior to the pandemic, we received visits with up to 60 people in one single day and up to 100 per week. As a result of the pandemic, we defined new strict protocol for receiving visitors in our lab. The restrictions from the national and local authorities on the maximum amount of participants at indoor events and the requirement of a minimum 1 m distance at all time put a limit on the number of people at a same time in our facilities at any given time. This limit is after a careful consideration set to 11, so large groups need to be divided and have a hold area where they can have a comfortable distance between themselves. A risk area is the entry to the lab, so we organise entry to get visitors to enter and exit the lab in an organised fashion, keeping distance.

To address these challenges, we are moving towards hybrid events, with physical participations and streaming/participation in VR.

## Conclusion and future work

In this paper we presented the case of running a research and education XR lab in Norway and discussed the actions we were required to take as a consequence of measures to control the spread of COVID 19. The feedback from students using the lab for their projects shows that they received adequate support to complete their research despite the complex circumstances. The lesson for XR lab operators is that they will need to have the ability to pack, hand in and track equipment on a very short notice under the new pandemic circumstances. XR equipment is portable and powerful so quality work can be carried out outside XR labs if necessary. In order to continue research under similar circumstances as those experienced in the first half of 2020, researchers have to evaluate whether they can complete their research from home, taking the hardware with them, and how does it affect other researchers. It might not be enough for XR groups to individually adopt new practices, there might also be necessary to agree on ways to obtain comparable insight from other research groups when unable to recruit large groups of participants.

Given the current development, it is highly likely that we would not be able to operate our lab as we used to before the pandemic, even as restrictions are being relaxed. Therefore, we have developed a new strategy for running an XR lab for research, education and dissemination purposes, with strict hygiene procedures to provide confidence and safety to lab users. These guidelines might be useful for other university XR labs as well. While bringing along tremendous disruption, the pandemic also triggered innovation. Immersive technologies can support rich remote communication and collaboration, also in the context of teaching and research. Several classes, seminars and conferences have been recently moved to online VR platforms such as Virbela and AltspaceVR. These capabilities should be explored future, e.g. to facilitate research when the researchers need to take the equipment home, by supporting colleagues remotely. The authors are currently working on a project investigating how to support rich and interactive university teaching in the context of pandemic with XR, especially in practical subjects where online lectures are not sufficient. The authors have also designed a new course on XR for remote collaboration, to help newly unemployed with transition to the new normal and the new workplace reality.
